# CCR7 deficient inflammatory Dendritic Cells are retained in the Central Nervous System

**DOI:** 10.1038/srep42856

**Published:** 2017-02-20

**Authors:** Benjamin D. Clarkson, Alec Walker, Melissa G. Harris, Aditya Rayasam, Martin Hsu, Matyas Sandor, Zsuzsanna Fabry

**Affiliations:** 1 Departments of Pathology and Laboratory Medicine, School of Medicine and Public Health, University of Wisconsin-Madison, Madison, WI 53792, USA; 2 Graduate Training Programs of Cellular and Molecular Pathology, School of Medicine and Public Health, University of Wisconsin-Madison, Madison, WI 53792, USA; 3 Neuroscience, School of Medicine and Public Health, University of Wisconsin-Madison, Madison, WI 53792, USA.

## Abstract

Dendritic cells (DC) accumulate in the CNS during neuroinflammation, yet, how these cells contribute to CNS antigen drainage is still unknown. We have previously shown that after intracerebral injection, antigen-loaded bone marrow DC migrate to deep cervical lymph nodes where they prime antigen-specific T cells and exacerbate experimental autoimmune encephalomyelitis (EAE) in mice. Here, we report that DC migration from brain parenchyma is dependent upon the chemokine receptor CCR7. During EAE, both wild type and CCR7−/− CD11c-eYFP cells infiltrated into the CNS but cells that lacked CCR7 were retained in brain and spinal cord while wild type DC migrated to cervical lymph nodes. Retention of CCR7-deficient CD11c-eYFP cells in the CNS exacerbated EAE. These data are the first to show that CD11c^high^ DC use CCR7 for migration out of the CNS, and in the absence of this receptor they remain in the CNS *in situ* and exacerbate EAE.

CNS autoimmune diseases, including multiple sclerosis (MS), affect more than 2 million individuals worldwide. Abrogation of peripheral tolerance to CNS antigens is thought to be a major component of the etiology of these diseases, potentially contributing to both the initiation of disease and relapse as immune responses are elicited against secondary antigens^1^,^2^. Yet, little is known about the processes of antigen drainage from immune privileged organs such as the CNS during both steady-state and neuroinflammatory conditions. Antigen drainage from other tissues is thought to be dependent upon passive flux of soluble antigens and active migration of tissue antigen-presenting cells (APC), such as dendritic cells (DC), through lymphatics to draining lymph nodes (LN). In the CNS, a surprisingly dense network of lymphatic vessels in the dura matter, underlying the skull bones have been recently detected^3^,^4^. However, whether these vessels are for macromolecular drainage, or active DC migration is still controversial. It was proposed that the dura matter lymphatic vessels are critical for the absorption of macromolecules from the brain interstitial fluid and CSF^3^. Mice injected intracerebroventricularly with exogenous antigen develop humoral and cell-mediated immune responses in cervical lymph nodes cLNs;^5^. Likewise, in mice injected intracerebrally (i.c.) with ovalbumin (OVA) protein, CD8+ OVA-specific (OT-1) T cells are recruited to the CNS after first proliferating in CLN, demonstrating that afferent immunity is intact in the CNS and that antigens drain or are trafficked to CLN^6^. Similarly, we have shown that constitutive or induced expression of neural tissue-specific neoantigens elicits antigen-specific T cell proliferation in peripheral lymphoid tissues^7^.

In contrast to soluble antigen drainage, little is known about the extent or mechanisms of cell-mediated antigen drainage from the CNS. Using animal models such as experimental autoimmune encephalomyelitis (EAE) to model CNS autoimmunity, we and others have reported that CD11c+ DC accumulate within the brain and spinal cord during neuroinflammation^8^,^9^,^10^. We have also shown that i.c. injected OVA-loaded inflammatory bone marrow-derived (BM)DC express low levels of CCR7 and migrate to deep CLN where they prime OVA-specific T cell responses^11^. This migration could be blocked by pretreatment of BMDC with pertussis toxin, suggesting that it is not passive migration and is rather mediated by G protein-dependent motility found downstream of many chemokine receptors. Furthermore, we have shown that i.c. injection of MOG peptide-loaded BMDC prior to induction of EAE exacerbates disease^12^, suggesting that the number of CNS-infiltrating DC is rate-limiting in the initiation of EAE. It is still unknown whether the primary role of CNS-infiltrating DC is to transport neuroantigen to draining LN for T cell priming or to restimulate MOG-specific effector T cells *in situ* within developing CNS inflammatory lesions. Therefore, we sought to better understand the mechanisms of BMDC migration from brain to CLN and test the role of CCR7 in the brain-CLN axis of CD11c+ DC migration.

Research employing photo-labeling of dermal DC has demonstrated that the chemokine receptor CCR7 directs migratory DC in the skin and lung to draining LN^13^,^14^,^15^,^16^,^17^. CCR7 binds to two ligands, CCL19 and CCL21, which are distributed in lymphatic vessels and lymph nodes and are essential for maintaining LN architecture and cell distribution^18^,^19^,^20^. CCL21 expression was demonstrated in lymphoid vessels in the dura matter of the CNS^3^.

While CCR7 is chiefly noted for its role in lymphatic migration, it has also been shown to be present in tissue during chronic inflammation and ectopic lymphoneogenesis^21^,^22^,^23^, both of which are associated with CNS autoimmune diseases such as MS. In fact, CCL19 is elevated in the cerebral spinal fluid (CSF) of patients with MS^24^,^25^, acute optic neuritis, and other inflammatory diseases, and CCL19 levels correlate with total CSF leukocyte number^24^. Moreover, DC expressing CCR7 have been detected in CSF of MS patients^26^. These data strongly implicate DC expression of CCR7 in CNS diseases; however, it remains to be determined if CCR7 mediates DC migration out of the CNS to CLN. Here we report that CCR7 mediates CD11c+ cell migration from the CNS parenchyma to the meningeal lymphoid vessels and eventually to the deep cervical LN during neuroinflammation. In the absence of CCR7, DC are retained in the CNS and exacerbate neuroinflammation. Regulating DC migration out from the inflamed CNS may be a therapeutic target for MS and other chronic neuroinflammatory conditions.

## Materials and Methods

### Mice and bone marrow chimeras

C57BL/6 (H2^b^) wild-type (WT, stock #000664), CCR7deficient B6.129P2(C)-*Ccr7*^*tm1Rfor*^/J (CCR7−/−, stock #006621), B6.PL-Thy1^a^/CyJ (Thy1.1, stock# 000406), and B6.Cg-Tg(CAG-DsRed*MST)1Nagy/J (Dsred, stock #006051) transgenic mice were obtained from the Jackson Laboratory (Bar Harbor, ME). B6.Cg-Tg(Itgax-Venus)1Mnz/J (CD11c-eYFP) transgenic mice on the C57BL/6 background were a generous gift from Dr. Michel C. Nussenzweig (Rockefeller University, NY). C57BL/6-Tg (Tcra2D2, Tcrb2D2)1Kuch/J (2D2) T cell receptor-transgenic mice with MOG_35–55_-H2^b^-restricted CD4+ T cells were a gift from Dr. Vijay Kuchroo (BWH-HMS, Boston, MA). 2D2 mice were crossed with Thy1.1 mice to generate 2D2.Thy1.1 mice. All F1 offspring used in experiments were screened for TCR-(Vα3 Vβ11) and Thy1.1 expression by flow cytometry on immune cells isolated from blood. Standard PCR screening was used for CD11c-eYFP (tgc tgg ttg ttg tgc tgt ctc atc, ggg ggt gtt ctg ctg gta gtg gtc), and CCR7−/− mice (WT, ttc cta gtg cct atg ctg gct atg, ggc aat gtt gag ctg ctt agct atg; mutant, ggg tgg gat tag ata aat gcc tgc tct; reverse, ggc aat gtt gag ctg ctt gct ggt t). CD11c-eYFP mice were bred and backcrossed with congenic CCR7−/− mice to generate CCR7−/−CD11c-eYFP mice. For preparation of chimeric mice by bone marrow (BM) transplantation, WT mice were irradiated (950 rads), and injected with a mixture of BM cells from WT, CCR7^−/−^, CD11c-eYFP, or CCR7−/− CD11c-eYFP (10–25 × 10^6^, i.v.) mice 4–10 hours after irradiation. All animal procedures used in this study were conducted in strict compliance with the National Institutes of Health Guide for the Care and Use of Laboratory Animals and approved by the University of Wisconsin Center for Health Sciences Research Animal Care Committee.

### Induction of EAE

For EAE induction, 100 μg rodent myelin oligodendrocyte glycoprotein peptide (MOG_35–55_, MEVGWYRSPFSRVVHLYRNGK) emulsified in equal volume CFA supplemented with *M. tuberculosis* H37Ra (5 mg/ml, Difco, Detroit, MI) was injected subcutaneously in the scapular region of each mouse. MOG-CFA mixture was emulsified by sonication using an ultrasonic homogenizer (Model 300VT equipped with a titanium cup tip, Biologics Inc. Monassas, VA). Pertussis toxin (200 ng/mouse, i.p.) was injected on days 0 and 2 relative to immunization. Mice were monitored daily in a blinded manner and clinical scores were recorded as previously described^12^. Mice were scored from 0–5 with 0 = no weakness, 1 = flaccid tail, 2 = gait disturbance or hind limb weakness, 3 = hind limb paralysis and no weight bearing on hind limbs, 4 = hind limb and forelimb paresis and reduced ability to move around the cage, and 5 = moribund or dead. The mean daily clinical score and standard error of the mean were calculated for each group. The significance of differences was calculated by Student’s *t* and Wilcox tests.

### Intracerebral microinjection

For i.c. injection, mice were anesthetized with ketamine (90 mg/kg) − xylazine (10 mg/kg) mixture (20 μl/mouse, i.p.). BMDC (2.5 × 10^5^), fluorescent nanoparticles (4% W/V) together with CCL2 (200 ng) and LPS (150 ng) or equal volume (20 μl) PBS was injected into the right frontal lobe using an insulin syringe (28 g) attached to a penetrating depth controller as previously described^6^,^27^. The injection was restricted to the ventral-posterior region of the frontal lobe, and the penetrating depth of the syringe was 1.55 mm from the surface of the brain. For each i.c. injection, the solution was injected slowly, and then the syringe was held in place for an additional 60 seconds to reduce backfilling of injected solution^6^,^27^. In some experiments, LPS was applied to activate TLR4-MyD88 dependent maturation signals in the DCs to promote their upregulation of CCR7 and migration to cLNs.

### Histology

For fluorescent microscopy, CNS and peripheral lymphoid organ tissues were fixed overnight in 3% formalin/25% sucrose, embedded in optimal cutting temperature (O.C.T) Compound (Tissue-Tek Sakura, Torrance, CA), and stored at −80 °C. Cryosections (5–10 μm) were cut from O.C.T-embedded tissue samples, post-fixed for 20 minutes in ice-cold acetone, and washed with PBS (25–50 minutes). After blocking for 30 minutes with 40 μg/mL 2.4G2 mAb in FACS buffer (1% bovine serum albumin in PBS), sections were incubated with saturating concentrations of fluorochrome-labeled fluorescent mAbs against CD11c (HL-3), B220 (RA3-6B2), CD4 (RM4.5), or CD8a (53-6.7) for two hours at room temperature in FACS buffer. After 3 washes with PBS (5 minutes each), sections were mounted with ProLong Gold anti-fade reagent (Invitrogen, Carlsbad, CA) with DAPI. Fluorescent images were acquired at 40–400x with Picture Frame software (Optronics Inc.) using an Olympus BX41 microscope (Leeds Precision Instruments) equipped with a camera (Optronics Inc., Goleta, CA). For bright field microscopy, CNS tissues were post-fixed in 10% formalin and embedded in paraffin for sectioning (10 μm). Tissue sections were stained with H&E or luxol fast blue (LFB) to detect infiltrating cells or demyelination, respectively. Bright-field images were acquired at 40–400x final magnification with Q-Capture software using an Olympus BX40 microscope equipped with a Q-Color 3 camera (Olympus America Inc.). Digital images were processed and analyzed using Photoshop CS4 software (Adobe Systems). Color balance, brightness, and contrast settings were manipulated to generate final images. All changes were applied equally to entire image.

### Real-time PCR

Deep CLN were dissected and stored in RNAlater (Qiagen, Valencia, CA) at 4 °C until further use. Total RNA was extracted and purified with RNeasy Protect Mini Kit (Qiagen) according to the manufacturer’s instructions. For RT-PCR, 1 μg total RNA from each sample was reverse transcribed using Super Script II first strand complementary (c) DNA synthesis kit (Invitrogen). RT-PCR was performed on a Smart Cycler (Model SC 100-1, Cepheid) using the eYFP Taqman gene expression assay (6FAM-ttc aag tcc gcc atg ccc gaa-Tamra, cca cat gaa, gca gca gga ctt ggt gcg ctc ctg gac gta; Applied Biosystems, Foster City, CA). The data were normalized to an internal reference gene, GAPDH, assessed using the following primers (ctc tgc tcc tcc tgt tcg ac, agg ggt cta cat ggc aac tg). Cell number was quantified by interpolating onto a ΔCT standard curve generated from performing eYFP RT-PCR on CD11c-eYFP BMDC serially diluted into WT BMDC, keeping total cell number constant as previously described^28^.

### Mononuclear cell isolation

Isolation of immune cells from brains, spinal cords, lymph nodes, and spleens of mice was performed as previously described^12^. Briefly, following transcardial perfusion with heparinized-PBS, CNS and peripheral lymphoid organ tissues were removed from mice and weighed. Spleen and lymph nodes were gently dissociated between frosted slides and cells were collected in HBSS. Brains and spinal cords were finely minced, homogenized by passaging through an 18 gauge needle, and incubated with collagenase Type IV (1 mg/ml) and DNase (28 U/mL) at 37 °C for 45 minutes under continuous rotation and inversion. Samples were further homogenized by trituration and filtered through a 70 μm cell strainers. CNS cell suspensions were washed with HBSS, resuspended in 70% Percoll (Pharmacia, Piscataway, NY), and overlaid with 30% Percoll. The gradients were centrifuged at 2,500 rpm (625xg) for 30 minutes at 4 °C without brake. Mononuclear cells were collected from the gradient interface and washed once before further analysis.

### Intracellular cytokine staining and flow cytometry

For *ex vivo* recall responses, single-cell suspensions from various tissues were cultured for 5 hours at 37 °C in 10% FBS in RPMI 1640 media supplemented with GolgiStop (BD Biosciences, San Jose, CA) and either PMA (50 ng/mL) and ionomycin (1 mg/ml), MOG_35–55_ peptide (2–20 μg/mL) or anti-CD3 (1 μg/mL)/anti-CD28 (2 μg/mL). For immunofluorescent labeling, 10^6^ cells isolated from CNS and peripheral lymphoid organ tissues were incubated for 30 min on ice with saturating concentrations of fluorochrome-labeled mAbs with 40 μg/mL unlabeled 2.4G2 mAb to block non-specific binding to Fc receptors. Cells were washed 3 times with 1% BSA in PBS. For intracellular staining, cell suspensions were fixed and permeabilized overnight (4 °C) with Cytofix/Cytoperm solution (BD Biosciences). The next day, cells were washed with Perm/Wash Buffer (BD Biosciences) and stained with anti-IFNg and anti-IL-17 mAbs. Fluorochrome-labeled mAbs against CD45 (30-F11), CD11b (M1/70), CD11c (HL3), CD80 (16-10A1), CD86 (GL1), CD40 (3/23), PDL1(MIH5), PDL2 (Ty25), IA^b^ (AF6-120.1), B220 (RA3-6B2), CD4 (RM4.5), Vβ11 (RR3-15), Thy1.1 (OX7), CD8a (53-6.7), IFN-γ (XMG1.2), IL-17 (TC11-18H10), and appropriate isotype controls were purchased from BD Biosciences (Minneapolis, MN). Fluorchrome-labeled mAbs against CD45.1 (A20) and CD45.2 (104) were purchased from Ebioscience (San Diego, CA). Cell staining was acquired on a FACSCalibur or LSRII (BD Biosciences) and analyzed with FlowJo (Tree Star) software version 10.0.6.

### Bone marrow DC differentiation

BMDC were generated as previously described^11^,^29^. Briefly, BM cell suspensions obtained from femurs and tibias of C57BL/6 mice were resuspended in ammonium chloride potassium-containing ACK lysis buffer to remove erythrocytes, washed, and plated in RPMI 1640 with 10–20% FBS supplemented with 100 U/ml penicillin/streptomycin and 20 ng/ml GM-CSF. GM-CSF was titrated from supernatants of the GM-CSF-secreting X63 cell line (gift from Dr. A. Erdei, Eotvos University, Budapest, Hungary). Six days following GM-CSF cultures, the non-adherent and loosely adherent BMDC precursors were removed and re-plated in a new flask. BMDC were collected and used for experiments between 9 and 13 days of culture. Surface expression of CD11b, CD11c, MHC II, CD80 and CD86 were confirmed by flow cytometry. For antigen pulse, BMDC were cultured with MOG_35–55_ peptide (10 μg/ml) and LPS (500 ng/mL) for 4 hours. After pulsing, cells were washed extensively before use.

### Statistical analyses

Results are given as means plus or minus one standard deviation. Multiple comparisons were made using one-way ANOVA or Kruskall-Wallis non-parametric ANOVA (EAE clinical scores). Log rank (Mantel-Cox) test was used for time to event comparisons. Bonferroni correction of threshold was used to account for multiple comparisons of log-rank tests, with P values < 0.025 being considered significant for these analyses. As appropriate, two-sided Student’s *t-*test analysis was used to compare measures made between two groups. *P* values < 0.05 were considered significant.

## Results

### CCR7^+/+^ but not CCR7^−/−^ BMDC can be recovered from deep CLN after i.c. injection

**Figure 1:**
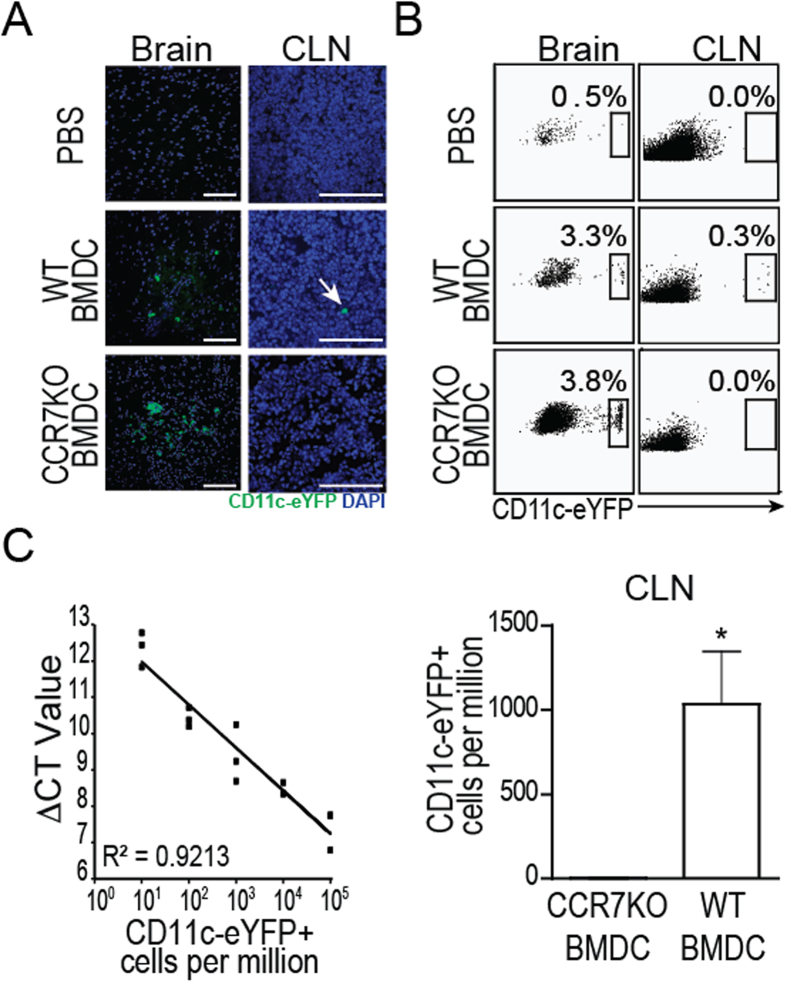
WT but not CCR7 KO i.c. injected DC migrate from brain to cervical lymph nodes. (**A**) Fluorescent micrographs of CD11c-eYFP DC one day post injection in cerebral cortex and 7 days later in CLN. Arrow indicates representative CD11c-eYFP DC in cervical lymph node. Scale bars represent 200 microns. (**B**) Flow cytometry plots show CD11c eYFP DC in brain, CLN, and spleen 7 days after intracerebral microinjection. (**C)** Quantification of CD11c-eYFP cells in CLN 7 days post i.c. injection. Cell counts were calculated by interpolation of ΔCT values from real-time PCR using a standard curve for EYFP transcript developed by serially diluting CD11c-EYFP DC into WT DC. *p < 0.05, student’s t test. Data are representative of 3 independent experiments with n = 9 per group.

CCR7 directs migratory DC in the skin and lung to draining LN^13^,^14^,^15^,^16^,^17^. In order to test whether CCR7 contributes to DC migration from the brain parenchyma to the CLN, we backcrossed B6 congenic DC-reporter mice expressing enhanced yellow fluorescent protein downstream of the CD11c promoter (CD11c-eYFP) with congenic CCR7 deficient (−/−) mice. Using CCR7−/− CD11c-eYFP reporter mice and the parent CD11c-eYFP CCR7 sufficient (+/+) mouse line, we generated DC by culturing BM cells with GM-CSF and confirmed that CCR7 deficient and sufficient cells were comparable for costimulatory molecule expression and antigen presenting capability (Fig. S1). We microinjected equal numbers of LPS maturated CCR7+/+CD11c-eYFP and CCR7−/− CD11c-eYFP BMDC into the brain of wild-type (WT) recipients and measured eYFP cell accumulation in the deep CLN^6^,^27^. LPS was used to activate TLR4-MyD88 dependent maturation signals in the DCs to promote their upregulation of CCR7. Control animals received equal volume i.c. injections of PBS. Fluorescent micrographs of the recipient brains 1 day post injection revealed the presence of CD11c-eYFP+ cells at injection sites in all animals except PBS injected controls. Using fluorescent microscopy and flow cytometry, we detected CD11c-eYFP+ cells in deep CLN of animals receiving CCR7+/+CD11c-eYFP+ BMDC but not those receiving CCR7−/− CD11c-eYFP+ BMDC at 4 and 7 days after injection (day 7 shown in Fig. 1A,B). Flow cytometry also confirmed that these cells were not migrating to lymphoid tissues via the blood stream, as they were absent from blood or spleen cells (data not shown).

To further quantify eYFP reporter cell accumulation in the deep CLN, we performed RT-PCR for eYFP transgene expression from tissue of CLN. We used a standard curve based on serial dilution of eYFP-expressing BMDC in eYFP- WT BMDC^28^ for quantifying CD11c cells numbers. We confirmed that CCR7+/+CD11c-eYFP+ cells accumulated in deep CLN and by day 7 were approximately 400x more abundant than CCR7−/− CD11c-eYFP+ cells (Fig. 1C). These results show for the first time that, similarly to skin and lung tissues, CD11c^high^ DC cell migration from brain to CLN depends upon CCR7.

Next, we asked whether ongoing neuroinflammation would modify the migration of CNS-infiltrating CD11c+ cells from brain to CLN, and whether this process would depend upon CCR7 regulated mechanisms. To this end, we optimized a protocol for i.c. delivery of fluorescent nanoparticles and adjuvant (200 ng CCL2 and 150 ng LPS) to attract immune cells to the injection site based on previous protocols^30^. Using this protocol we observed strong immune cell recruitment to the injected hemisphere within 24 hours. We also measured CD11c+ cell recruitment to the cerebral injection site, in parallel with the engulfment of fluorescent beads, and trafficking of bead positive cells to the CLN by flow cytometry in CCR7+/+CD11c-eYFP + and CCR7−/− CD11c-eYFP+ mice. We found marked recruitment of myeloid DC to the ipsilateral hemisphere of i.c. injected mice within 24 hours of injection (data not shown). We also noticed that in WT mice, the number of nanoparticles-containing CD11c-eYFP+ cells increased 1–7 days post injection in the deep CLN (Fig. S2). In contrast, in CCR7−/− mice we saw significantly lower level of accumulation of nanoparticles-containing CD11c-eYFP+ cells in the deep CLN (Fig. S2). Taken together these data suggested that CCR7 promotes cell-mediated drainage of insoluble particles from CNS and contributes to CD11c+ cell migration from brain to CLN.

### CCR7^−/−^ DC accumulate in the CNS but do not reach the CLN during EAE

**Figure 2:**
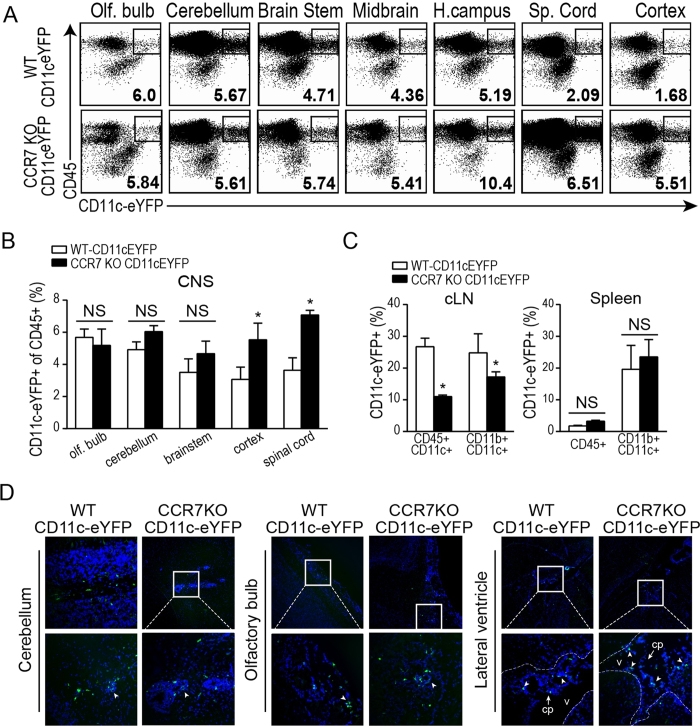
CCR7 KO CD11c+ DC accumulate in cortical brain tissue and spinal cord during EAE. (**A**) Representative dot plots from 3 independent experiments with 3–4 mice per group show frequency of CD45 + CD11c-eYFP+ cells among total CD45+CD11b+ cells isolated from different brain areas of CCR7−/− CD11c-eYFP and CCR7+/+CD11c-eYFP recipient bone marrow chimeric mice with EAE (Day 12). Column graphs show CD11c-eYFP+ cell frequency among indicated population of cells isolated from the indicated CNS anatomical regions **(B)** or CLN and spleen **(C)** of CCR7−/− CD11c-eYFP and CCR7+/+CD11c-eYFP recipients with EAE (Day 12). (**D)** Fluorescent micrographs of DAPI-stained saggital brain sections showing CD11c-eYFP+ cell accumulation in cerebellum, olfactory bulb, and lateral ventricles of CCR7−/− CD11c-eYFP and CCR7+/+CD11c-eYFP recipient bone marrow chimeric mice with EAE (Day 12). *p < 0.05, student’s t test.

We next sought to determine if CNS-infiltrating CCR7−/− CD11c+DC were differentially retained in the CNS during EAE compared to CNS-infiltrating CCR7+/+CD11c+DC. We generated mixed BM chimeras by injecting irradiated WT recipients with BM cells from WT mice mixed 1:1 with either CCR7+/+CD11c-eYFP or CCR7−/− CD11c-eYFP BM cells. After reconstitution and recovery, we induced EAE by MOG_35–55_/CFA immunization. As expected, due to the presence of a partially WT hematopoietic system, we observed no difference in EAE disease progression between CCR7+/+CD11c-eYFP and CCR7−/− CD11c-eYFP BM recipients (data not shown). At days 8, 12, and 16 we isolated immune cells from peripheral lymphoid organ tissues and gross-dissected CNS tissue regions to compare the accumulation of CCR7+/+CD11c-eYFP+ cells and CCR7−/− CD11c-eYFP+ cells by flow cytometry (gating shown in Fig. 2A). Relative to all CD45 + immune cells, CCR7+/+and CCR7−/− CD11c-eYFP+ cells were present at similar levels in spleen (Fig. 2C). During preclinical EAE (Day 8), CCR7−/− CD11c-eYFP+ cells were less abundant in the CNS than their WT counterparts (data not shown). However, by Day 12 of EAE CCR7−/− CD11c-eYFP+ cells had accumulated to similar levels in olfactory bulb, lateral ventricles, hippocampus, cerebellum and brain stem compared to CCR7+/+CD11c-eYFP+ cells as measured by flow cytometry (Fig. 2B) and fluorescent microscopy (Fig. 2D). In contrast, we measured higher frequency of CCR7−/− CD11c-eYFP+ cells among CD45+ cells in both spinal cord and cerebral cortex at day 12 (Fig. 2B). CCR7−/− CD11c.eYFP+ cells continued to outnumber WT CD11c.eYFP+ cells in spinal cord and cortex at day 16 at which time their relative abundance in olfactory bulb and cerebellum was reduced (data not shown). These changes were accompanied by a decreased accumulation of CCR7−/− CD11c-eYFP+ cells in CLN (Fig. 2C). This further supports that CCR7-deficient inflammatory CD11c+ dendritic cells are retained in different areas of the CNS during neuroinflammation, while CCR7 expressing dendritic cells can migrate out of the CNS.

### CCR7 deficient CD11c+ DC in the CNS exacerbate EAE

We have previously demonstrated that i.c. microinjection of mature MOG-loaded BMDC can exacerbate EAE induced neuroinflammation^12^. However, whether this effect is dependent upon the migration of injected BMDC from brain to CLN for T cell priming is unknown. To generate mature MOG-loaded WT and CCR7−/− DC, BMDC were pulsed with MOG_35–55_ peptide (10 μg/mL) for 4 hours and matured with LPS (500 ng/ml). BMDC maturation was confirmed by measuring upregulation of surface markers before and after LPS treatment (Fig. S1). After washing extensively, mature MOG-loaded WT or CCR7−/− BMDC were injected i.c. into WT mice 5 days prior to EAE induction.

**Figure 3:**
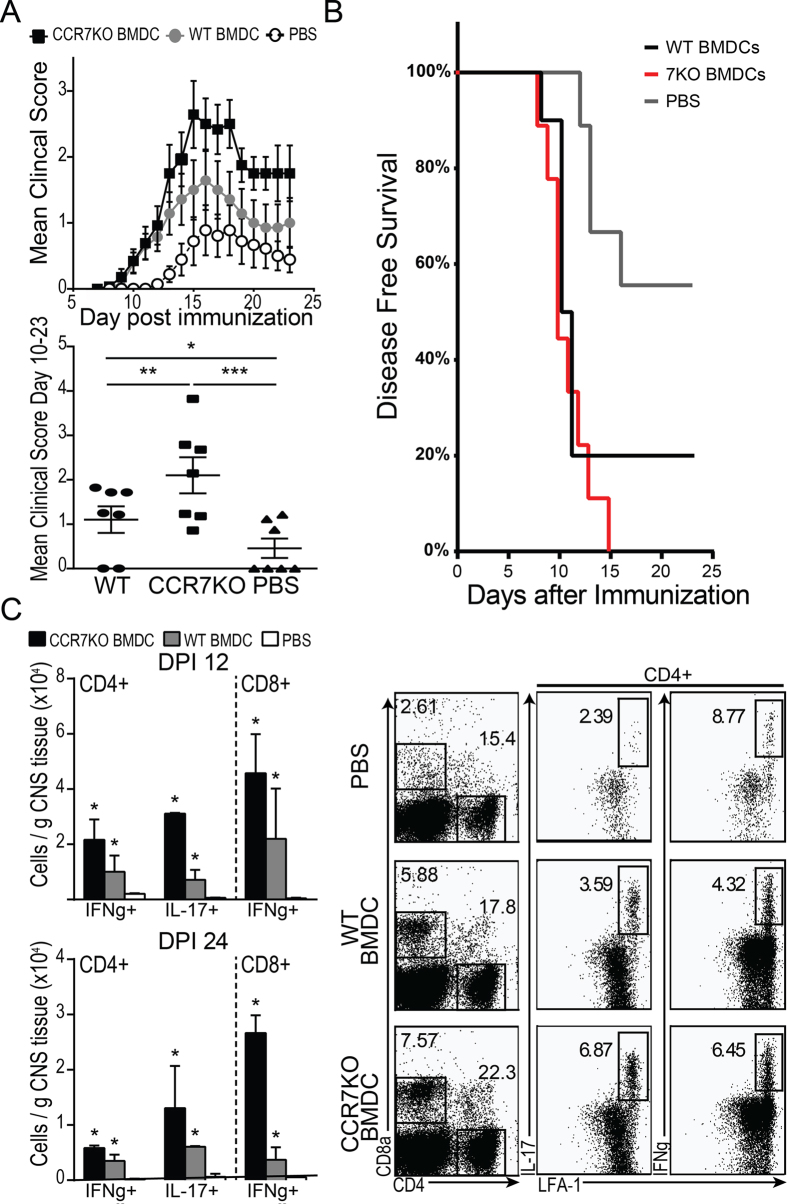
Retention of CCR7-deficient DC in the CNS further exacerbate EAE compared to WT DC. (**A)** Mean clinical scores of WT mice injected intracerebrally with MOG-pulsed WT DC, MOG-pulsed CCR7 KO DC, or PBS 5 days before EAE induction. *p < 0.05, **p < 0.001, ***p < 0.0001, one-way ANOVA. (**B**) Kaplan Meier plot shows disease free survival among mice receiving i.c. injections of PBS, WT DC, or CCR7 KO DC. (**C**) FACS plots show frequency of IL-17 and IFNγ producing cells among CD4+ T cells isolated from day 12 EAE brains of WT mice i.c. injected 17 days prior with MOG-pulsed WT DC, MOG-pulsed CCR7 KO DC, or PBS (quantified on left). *p < 0.05, one-way ANOVA. Data are representative of 3 independent experiments with 3–4 mice per group.

We observed earlier clinical onset (Fig. 3A,B, log rank p < 0.05) and higher peak and mean clinical score in mice i.c. injected with WT BMDC compared to PBS controls. We saw a similar acceleration of EAE clinical onset in mice receiving CCR7−/− BMDC (Fig. 3A,B, log rank p < 0.001), with significantly higher mean clinical score in these mice compared to mice receiving WT BMDC or PBS control injection. This was accentuated at later time points after initial clinical onset (day 10–23). We also saw an increase in the frequency of effector helper T cells (CD4+ LFA-1+) producing IFNγ (Th1) or IL-17 (Th17) in response to *ex vivo* restimulation with MOG_35–55_ peptide in BMDC recipients compared to PBS injected controls (Fig. 3C). Interestingly, levels of IFNγ and IL-17 producing CD4+ T cells in the CNS of CCR7−/− BMDC recipients were higher compared to WT BMDC recipients.

**Figure 4:**
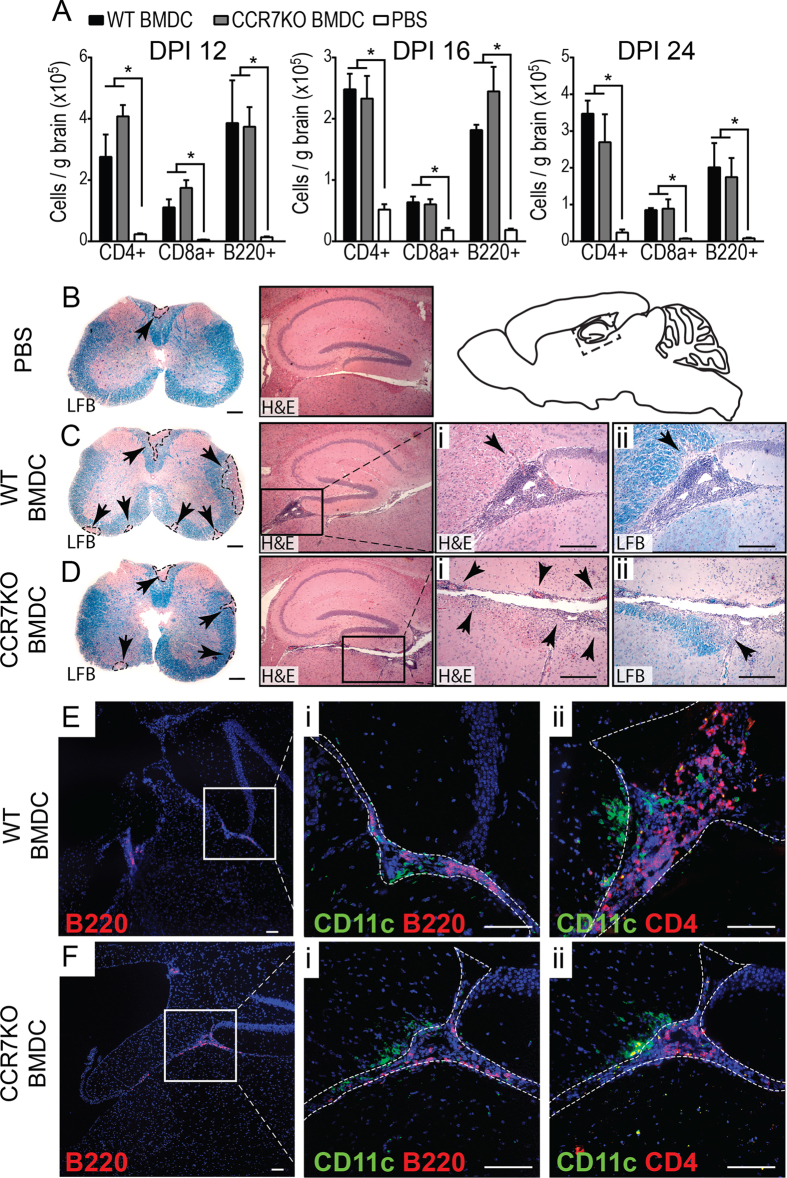
Perivascular lymphocytes accumulation in EAE brain following i.c. injection of WT or CCR7 KO BMDC_MOG_. (**A**) Column graphs show total number (per gram tissue) and frequency (total brain cells) of brain-infiltrating T cells (CD4 + or CD8+) and B cells (B220+) in WT mice with EAE (DPI 12, 16, 24) that were i.c. injected as indicated. *p < 0.05 one-way ANOVA, no significant differences were observed between WT and CCR7 KO BMDC recipients. H&E and LFB stained day 24 EAE saggital brain and cross sectional spinal cord sections from WT mice injected with PBS **(B)**, MOG-pulsed WT DC **(C)** or MOG-pulsed CCR7 KO DC **(D)** 5 days before EAE induction. Black arrowheads indicate areas of cellular infiltration into parenchyma **(Ci, Di)** and demyelination in the brain **(Cii, Dii)** and spinal cord **(B–D** left). Fluorescent micrographs of antibody stained day 24 EAE brains cryosections from WT mice injected with MOG-pulsed WT DC **(E)** or MOG-pulsed CCR7 KO DC **(F)**. Data are representative of 3 independent experiments with n = 3–5 mice per group. Scale bar 50 microns.

Accordingly, mice that received i.c. injection of WT or CCR7−/− BMDC had more lymphocytes accumulating in brain and spinal cord tissues throughout EAE clinical disease (EAE day 12, 16, and 24) compared to PBS injected controls. In particular, we observed a consistent increase in the frequency of CD4+ and CD8+ T cells as well as B220+ B cells within the brains of BMDC recipients (Fig. 4A), which were frequently found adjacent to CD11c+ cells in extra-parenchymal spaces near the injection site (Fig. 4E,F). By histological methods, we saw profound increases in leukocyte accumulation in the brain of mice receiving i.c. injections of MOG-pulsed BMDC compared to PBS injected controls (Fig. 4B–D). Intriguingly, we observed a striking difference in the leukocyte distribution between WT BMDC and CCR7−/− BMDC recipients. In mice receiving WT BMDC, leukocytes accumulated in the CSF-filled ventricular and subarachnoid spaces of deep sulci circa CA3 region of the hippocampus, dentate gyrus, and the anterior portion of the superior colliculus. Of note, most of these leukocytes did not infiltrate into the brain parenchyma, which is delineated from the CSF filled spaces by the pia mater, glia limitans, and ependymal wall (Fig. 4Ci). By contrast, in recipients of CCR7−/− BMDC, leukocytes were again distributed along the pial and ependymal surfaces, but showed marked infiltration into the periventricular brain parenchyma (Fig. 4Di). This increased infiltration in CCR7−/− BMDC recipients was accompanied by a slight increase in demyelinating lesions in the brain that corresponded with the location of increased leukocyte infiltration (Fig. 4Dii). Taken together, these data support that BMDC migration from brain to CLN depends on CCR7, and retention of CCR7-deficient CD11c^high^ cells in the CNS promote neuroinflammation.

## Discussion

This study is the first to show that DC migration from the brain to CLN is mediated by CCR7. In the absence of CCR7, DC are retained in the brain and contribute to the increase of EAE clinical scores. Exacerbation of EAE is correlated with increased frequency of IFN-γ or IL17 producing CD4 T cells and IFN- γ producing CD8 T cells in the CNS. These findings have the following implications: first, infiltrating DC could migrate to the cerebral lymphoid vessels and to the CLN via a CCR7 dependent mechanism and second, DC retention in the brain contributes to the acceleration of neuroinflammation.

Previous studies have demonstrated an important role for CCR7 in the migration of DC from different tissues to draining lymph nodes under both steady-state and inflammatory conditions^31^,^32^,^33^,^34^. Here, for the first time, we report the critical importance of CCR7 in migration of DC from the brain to CLN. We injected high numbers of CCR7 deficient or WT DC into the brain parenchyma mimicking inflammatory conditions within the CNS^11^,^27^,^35^ and tested DC accumulation in the deep CLN. We show that CCR7-deficient DC retention within the CNS exacerbates EAE.

Potential routes for immune cell migration out of the CNS are still subject to investigations. It was suggested that drainage of soluble CNS antigens might occur along vascular basement membranes and perivascular spaces that are contiguous with the CSF-filled subarachnoid space^36^,^37^,^38^. From there, cells might migrate along cranial nerves into tissues with normal lymphatic drainage. In particular, it has been suggested that immune cells might migrate along the olfactory projections across the cribriform plate into nasal mucosa^5^,^36^,^37^,^39^,^40^,^41^,^42^. As proof of principle, i.c.-injected GFP+ monocytes (which are precursors for certain DC populations) were detected in deep CLN 7 days after injection. Fluorescent microscopy on decalcified mouse head whole mounts revealed that GFP+ cells accumulated along the alveus of the hippocampus and ventral horn of the lateral ventricle. GFP+ cells subsequently accumulated on the ventral surface of the olfactory bulb, where they were found along olfactory nerve projections through the cribriform plate into nasal mucosa, which drains to CLN^43^,^44^. More recently, lymphatic vessels in the dura matter, underlying the skull bones have been described in more detail^3^,^4^. Intraparenchymal injection of tracers showed that the interstitial fluid is cleared directly from the subarachnoid space to the dura matter lymphoid vasculature^3^. More importantly, CD11c expressing cells were found in the dural lymphoid vessels of naïve mice^4^; however, the mechanism of cell migration into these vessels has not been explored in the CNS. We propose here that CCR7-CCR7-ligand axis is important in this process.

The expression patterns of CCR7 ligands at different stages of EAE offer insight that may be useful for understanding the CNS-CLN migratory axis. For example the CCR7 ligand, CCL19, is expressed at the RNA level in cerebellum and brain stem tissue during preclinical EAE, where it is upregulated during disease onset and during relapse^45^. More recently, Krumbholz *et al*. observed elevated levels of CCL19 in CSF of MS patients, where it correlated with IgG levels^25^, corroborating a previous report where elevated levels of either CCR7 ligand (CCL19 and CCL21) in MS patients correlated weakly with total cell counts^24^. These studies suggest a role for CCR7 and its ligands in immune cell migration and/or retention in CSF and neuronal parenchyma during CNS autoimmunity. The former hypothesis is reinforced by our data showing reduced accumulation of CCR7−/− DC in brain draining LN during EAE and reduced recovery rate of i.c. injected CCR7−/− BMDC from CLN.

Which phase(s) of the CNS-CLN DC migratory process require CCR7 remains unclear. As mentioned above, previous reports have demonstrated an important role for CCR7 in directional migration of DC through lymphatic vessels draining skin, lung and other tissues^31^,^32^,^33^. Our data showing that CCR7−/− DC accumulate to higher levels in brain cortex and spinal cord compared to WT counterparts indicate that CCR7-dependent emigration might be halted in these tissues. In support of this, data from a recent comprehensive digital gene expression library^46^,^47^ indicate that in healthy mice CCL21 is expressed in cerebral cortex with higher expression in the frontal cortex, suggesting it might play a role in directional DC migration through this region. Finally, the level of the soluble chemoattractant CCL19 in CSF may play a role in the trafficking of DC between parenchyma and CSF filled compartments, which have been shown to readily drain to CLN^48^ (reviewed in refs 36,37).

Here we found that artificially increasing the number of mature CNS DC accelerates EAE clinical onset by promoting cytokine production by co-infiltrating effector T cells—not by increasing the amount of T cell priming through cell-mediated drainage of CNS antigens to CLN. In fact we found that abolishing CCR7-dependent migration of antigen-loaded mature BMDC from brain to CLN was associated with increased CNS inflammation and disease severity. Similarly, it was shown that pharmacological inhibition of DC emigration from CNS with fingolimod worsens EAE clinical disease, and blocking DC emigration from brain under steady-state conditions can induce markedly increased frequency of spontaneous demyelinating disease in 2D2 TCR-transgenic mice with MOG_35–55_-specific CD4+ T cells^42^. This suggests an important role for CNS emigrant DC in regulating CNS immunity.

In our studies, LPS-induced maturation of BMDCs was chosen based on our previous finding with this maturation paradigm. However, in the context of MS it is thought that factors present in the CNS inflammatory milieu elicit DC maturation, which may differ significantly from LPS-induced DC maturation. Additionally, it must be noted that the observed effects on EAE disease course were observed on a mild EAE background, which is likely to be more responsive to factors that exacerbate disease course. Here we focused our studies on the recruitment and retention of DC to the brain during preclinical (day 8), early (day 12), and peak (day 16) disease as we have previously described the critical role of CNS DC during EAE onset (12,49). We have also observed that CNS DC continue to accumulate in the brain and especially in spinal cord tissues^50^ during chronic phases of EAE (Day20+; unpublished observations). Future studies will elaborate on the potentially distinct role of CNS emigrant DCs during chronic neuroinflammation.

Our data show that CCR7−/− BMDC that are preferentially retained in the CNS, promote CNS T cell activation and enable greater invasion of CNS parenchyma by inflammatory cells. CNS-infiltrating encephalitogenic T cells must re-encounter their cognate antigen on CNS APC before carrying out effector functions. Thus, our findings reinforce the idea that during early EAE the number of myelin-presenting APC (especially DC) in the CNS is a limiting factor for disease progression. This would be consistent with a previous report where CCR7−/− DC accumulated in lung during fungal infection and promoted enhanced immunity and fungal clearance^34^. Hartigan *et al*. argued that increased DC accumulation in the target tissue was necessary for robust T cell activation as they observed transferring DC to the target tissue promoted immunity and clearance. In striking similarity, they also observed that transferring CCR7−/− DC further promoted protective immunity, potentially due to increased local retention of DC in lung tissue.

We also demonstrated that i.c. injected BMDC promote lymphocyte recruitment to the CNS, and that one of the first signs of this increased recruitment is the preferential recruitment (as fraction of total brain lymphocytes) of B220+ B cells to brain. This effect was seen at day 12 of EAE, and it persisted at all later time points. Interestingly, when the clinical score of mice i.c. injected with CCR7−/− or WT BMDC first begins to statistically diverge (DPI 15–16), we saw higher B cell recruitment (both total recruitment and as a percent of brain lymphocytes) in CCR7−/− BMDC injected mice. These B cells were mostly found in extraparenchymal clusters closely associated with DC, which are reminiscent of meningeal B cell follicles and ectopic lymphoid tissues described in MS and EAE^46^,^51^,^52^,^53^,^54^,^55^,^56^,^57^,^58^,^59^. Ectopic lymphoid tissues are present in many autoimmune and chronic inflammatory conditions, including MS where they are thought to play a role in cortical demyelination and pathology^55^,^56^. CCR7 deficiency has been associated with development of tertiary lymphoid structures in gut mucosa^60^, and DC have an established role in ectopic-lymphoneogenesis and lymph angiogenesis through CXCL-13 and VEGF-C production^52^,^61^,^62^,^63^. Thus, it’s possible that when tissue infiltrating DC fail to migrate to draining LN they instead support development of tertiary lymphoid tissue adjacent to the inflamed organ tissue. To further investigate the possible role of CNS DC in ectopic-lymphoneogenesis, it would be valuable to determine the potential of i.c. injected CXCL13 deficient and VEGF-C haplo-insufficient BMDC to promote B cell recruitment to the CNS and follicle structure formation in the CNS in the context of EAE.

In summary, we show that CCR7 regulates DC migration from brain to CLN. Due to their high immunomodulatory potential, DC are crucial targets in T cell-mediated chronic neuroinflammatory conditions, such as MS. Failure of cell-mediated CNS antigen drainage has recently been implicated in the initiation of CNS autoimmunity using mouse models^42^. Clarifying the role of DC mediated antigen drainage and *in situ* T cell restimulation will help us identify cellular and molecular mechanisms that contribute to neuroinflammation.

## Additional Information

**How to cite this article**: Clarkson, B. D. *et al*. CCR7 deficient inflammatory Dendritic Cells are retained in the Central Nervous System. *Sci. Rep.* **7**, 42856; doi: 10.1038/srep42856 (2017).

**Publisher's note:** Springer Nature remains neutral with regard to jurisdictional claims in published maps and institutional affiliations.

## Supplementary Material

Supplementary Data File

## References

[b1] Goverman J. Autoimmune T cell responses in the central nervous system. Nature reviews. Immunology 9, 393–407, doi: 10.1038/nri2550 (2009). PMC2813731 19444307

[b2] Goverman J. M. Immune tolerance in multiple sclerosis. Immunological reviews 241, 228–240, doi: 10.1111/j.1600-065X.2011.01016.x (2011). 21488900 PMC3088997

[b3] Aspelund A. . A dural lymphatic vascular system that drains brain interstitial fluid and macromolecules. J Exp Med 212, 991–999, doi: 10.1084/jem.20142290 (2015). 26077718 PMC4493418

[b4] Louveau A. . Structural and functional features of central nervous system lymphatic vessels. Nature 523, 337–341, doi: 10.1038/nature14432 (2015). 26030524 PMC4506234

[b5] Cserr H. F., Harling-Berg C. J. & Knopf P. M. Drainage of brain extracellular fluid into blood and deep cervical lymph and its immunological significance. Brain Pathol 2, 269–276 (1992). 1341962 10.1111/j.1750-3639.1992.tb00703.x

[b6] Ling C., Sandor M. & Fabry Z. *In situ* processing and distribution of intracerebrally injected OVA in the CNS. Journal of neuroimmunology 141, 90–98 (2003). 12965258 10.1016/s0165-5728(03)00249-2

[b7] Harris M. G. . Immune privilege of the CNS is not the consequence of limited antigen sampling. Sci Rep 4 (2014). 10.1038/srep04422 PMC3961746 24651727

[b8] Fischer H. G. & Reichmann G. Brain dendritic cells and macrophages/microglia in central nervous system inflammation. J Immunol 166, 2717–2726 (2001). 11160337 10.4049/jimmunol.166.4.2717

[b9] Bailey S. L., Schreiner B., McMahon E. J. & Miller S. D. CNS myeloid DCs presenting endogenous myelin peptides ‘preferentially’ polarize CD4+ T(H)-17 cells in relapsing EAE. Nat Immunol 8, 172–180, doi: 10.1038/ni1430 (2007). 17206145

[b10] Serafini B., Columba-Cabezas S., Di Rosa F. & Aloisi F. Intracerebral recruitment and maturation of dendritic cells in the onset and progression of experimental autoimmune encephalomyelitis. The American journal of pathology 157, 1991–2002, doi: 10.1016/S0002-9440(10)64838-9 (2000). 11106572 PMC1885753

[b11] Karman J., Ling C., Sandor M. & Fabry Z. Dendritic cells in the initiation of immune responses against central nervous system-derived antigens. Immunology letters 92, 107–115, doi: 10.1016/j.imlet.2003.10.017 (2004). 15081534

[b12] Zozulya A. L. . Intracerebral dendritic cells critically modulate encephalitogenic versus regulatory immune responses in the CNS. The Journal of neuroscience: the official journal of the Society for Neuroscience 29, 140–152, doi: 10.1523/JNEUROSCI.2199-08.2009 (2009). 19129392 PMC2942091

[b13] Gunn M. D. . Mice lacking expression of secondary lymphoid organ chemokine have defects in lymphocyte homing and dendritic cell localization. J Exp Med 189, 451–460 (1999). 9927507 10.1084/jem.189.3.451 PMC2192914

[b14] Saeki H., Moore A. M., Brown M. J. & Hwang S. T. Cutting edge: secondary lymphoid-tissue chemokine (SLC) and CC chemokine receptor 7 (CCR7) participate in the emigration pathway of mature dendritic cells from the skin to regional lymph nodes. J Immunol 162, 2472–2475 (1999). 10072485

[b15] Jakubzick C., Tacke F., Llodra J., van Rooijen N. & Randolph G. J. Modulation of dendritic cell trafficking to and from the airways. J Immunol 176, 3578–3584 (2006). 16517726 10.4049/jimmunol.176.6.3578

[b16] Johnson L. A. & Jackson D. G. Inflammation-induced secretion of CCL21 in lymphatic endothelium is a key regulator of integrin-mediated dendritic cell transmigration. International immunology 22, 839–849, doi: 10.1093/intimm/dxq435 (2010). 20739459

[b17] Tal O. . DC mobilization from the skin requires docking to immobilized CCL21 on lymphatic endothelium and intralymphatic crawling. J Exp Med 208, 2141–2153, doi: 10.1084/jem.20102392 (2011). 21930767 PMC3182054

[b18] Forster R. . CCR7 coordinates the primary immune response by establishing functional microenvironments in secondary lymphoid organs. Cell 99, 23–33 (1999). 10520991 10.1016/s0092-8674(00)80059-8

[b19] Ohl L. . Cooperating mechanisms of CXCR5 and CCR7 in development and organization of secondary lymphoid organs. J Exp Med 197, 1199–1204, doi: 10.1084/jem.20030169 (2003). 12732661 PMC2193963

[b20] Fukuyama S. . Cutting edge: Uniqueness of lymphoid chemokine requirement for the initiation and maturation of nasopharynx-associated lymphoid tissue organogenesis. J Immunol 177, 4276–4280 (2006). 16982861 10.4049/jimmunol.177.7.4276

[b21] Page G., Lebecque S. & Miossec P. Anatomic localization of immature and mature dendritic cells in an ectopic lymphoid organ: correlation with selective chemokine expression in rheumatoid synovium. J Immunol 168, 5333–5341 (2002). 11994492 10.4049/jimmunol.168.10.5333

[b22] Grant A. J. . Hepatic expression of secondary lymphoid chemokine (CCL21) promotes the development of portal-associated lymphoid tissue in chronic inflammatory liver disease. The American journal of pathology 160, 1445–1455, doi: 10.1016/S0002-9440(10)62570-9 (2002). 11943728 PMC1867219

[b23] Timmer T. C. . Inflammation and ectopic lymphoid structures in rheumatoid arthritis synovial tissues dissected by genomics technology: identification of the interleukin-7 signaling pathway in tissues with lymphoid neogenesis. Arthritis and rheumatism 56, 2492–2502, doi: 10.1002/art.22748 (2007). 17665400

[b24] Pashenkov M., Soderstrom M. & Link H. Secondary lymphoid organ chemokines are elevated in the cerebrospinal fluid during central nervous system inflammation. Journal of neuroimmunology 135, 154–160 (2003). 12576236 10.1016/s0165-5728(02)00441-1

[b25] Krumbholz M. . CCL19 is constitutively expressed in the CNS, up-regulated in neuroinflammation, active and also inactive multiple sclerosis lesions. Journal of neuroimmunology 190, 72–79, doi: 10.1016/j.jneuroim.2007.07.024 (2007). 17825430

[b26] Kivisakk P. . Expression of CCR7 in multiple sclerosis: implications for CNS immunity. Annals of neurology 55, 627–638, doi: 10.1002/ana.20049 (2004). 15122702

[b27] Karman J., Ling C., Sandor M. & Fabry Z. Initiation of immune responses in brain is promoted by local dendritic cells. J Immunol 173, 2353–2361 (2004). 15294948 10.4049/jimmunol.173.4.2353

[b28] Schreiber H. A. . Inflammatory dendritic cells migrate in and out of transplanted chronic mycobacterial granulomas in mice. The Journal of clinical investigation 121, 3902–3913, doi: 10.1172/JCI45113 (2011). 21911937 PMC3195456

[b29] Inaba K. . Generation of large numbers of dendritic cells from mouse bone marrow cultures supplemented with granulocyte/macrophage colony-stimulating factor. J Exp Med 176, 1693–1702 (1992). 1460426 10.1084/jem.176.6.1693 PMC2119469

[b30] Puntambekar S. S. . LPS-induced CCL2 expression and macrophage influx into the murine central nervous system is polyamine-dependent. Brain, behavior, and immunity 25, 629–639, doi: 10.1016/j.bbi.2010.12.016 (2011). PMC3081407 21237263

[b31] Jang M. H. . CCR7 is critically important for migration of dendritic cells in intestinal lamina propria to mesenteric lymph nodes. J Immunol 176, 803–810 (2006). 16393963 10.4049/jimmunol.176.2.803

[b32] Ohl L. . CCR7 governs skin dendritic cell migration under inflammatory and steady-state conditions. Immunity 21, 279–288, doi: 10.1016/j.immuni.2004.06.014 (2004). 15308107

[b33] Seth S. . CCR7 essentially contributes to the homing of plasmacytoid dendritic cells to lymph nodes under steady-state as well as inflammatory conditions. J Immunol 186, 3364–3372, doi: 10.4049/jimmunol.1002598 (2011). 21296980

[b34] Hartigan A. J., Westwick J., Jarai G. & Hogaboam C. M. CCR7 deficiency on dendritic cells enhances fungal clearance in a murine model of pulmonary invasive aspergillosis. J Immunol 183, 5171–5179, doi: 10.4049/jimmunol.0901027 (2009). 19783686

[b35] Karman J. . Dendritic cells amplify T cell-mediated immune responses in the central nervous system. J Immunol 177, 7750–7760 (2006). 17114446 10.4049/jimmunol.177.11.7750

[b36] Weller R. O., Galea I., Carare R. O. & Minagar A. Pathophysiology of the lymphatic drainage of the central nervous system: Implications for pathogenesis and therapy of multiple sclerosis. Pathophysiology: the official journal of the International Society for Pathophysiology/ISP 17, 295–306, doi: 10.1016/j.pathophys.2009.10.007 (2010). 19954936

[b37] Laman J. D. & Weller R. O. Drainage of cells and soluble antigen from the CNS to regional lymph nodes. J Neuroimmune Pharmacol 8, 840–856, doi: 10.1007/s11481-013-9470-8 (2013). 23695293 PMC7088878

[b38] Carare R. O., Hawkes C. A. & Weller R. O. Afferent and efferent immunological pathways of the brain. Anatomy, Function and Failure. Brain, behavior, and immunity 36, 9–14, doi: 10.1016/j.bbi.2013.10.012 (2014). 24145049

[b39] Yamada S., DePasquale M., Patlak C. S. & Cserr H. F. Albumin outflow into deep cervical lymph from different regions of rabbit brain. The American journal of physiology 261, H1197–1204 (1991). 1928403 10.1152/ajpheart.1991.261.4.H1197

[b40] Furukawa M., Shimoda H., Kajiwara T., Kato S. & Yanagisawa S. Topographic study on nerve-associated lymphatic vessels in the murine craniofacial region by immunohistochemistry and electron microscopy. Biomed Res 29, 289–296 (2008). 19129672 10.2220/biomedres.29.289

[b41] Weller R. O., Djuanda E., Yow H. Y. & Carare R. O. Lymphatic drainage of the brain and the pathophysiology of neurological disease. Acta neuropathologica 117, 1–14, doi: 10.1007/s00401-008-0457-0 (2009). 19002474

[b42] Mohammad M. G. . Immune cell trafficking from the brain maintains CNS immune tolerance. The Journal of clinical investigation 124, 1228–1241, doi: 10.1172/JCI71544 (2014). 24569378 PMC3934177

[b43] Kaminski M., Bechmann I., Kiwit J. & Glumm J. Migration of monocytes after intracerebral injection. Cell adhesion & migration 6, 164–167, doi: 10.4161/cam.20281 (2012). 22568987 PMC3427229

[b44] Kaminski M. . Migration of monocytes after intracerebral injection at entorhinal cortex lesion site. Journal of leukocyte biology 92, 31–39, doi: 10.1189/jlb.0511241 (2012). 22291210

[b45] Columba-Cabezas S., Serafini B., Ambrosini E. & Aloisi F. Lymphoid chemokines CCL19 and CCL21 are expressed in the central nervous system during experimental autoimmune encephalomyelitis: implications for the maintenance of chronic neuroinflammation. Brain pathology 13, 38–51 (2003). 12580544 10.1111/j.1750-3639.2003.tb00005.x PMC8095989

[b46] Aloisi F. & Pujol-Borrell R. Lymphoid neogenesis in chronic inflammatory diseases. Nature reviews. Immunology 6, 205–217, doi: 10.1038/nri1786 (2006). 16498451

[b47] Lein E. S. . Genome-wide atlas of gene expression in the adult mouse brain. Nature 445, 168–176, doi: 10.1038/nature05453 (2007). 17151600

[b48] Mathieu E., Gupta N., Macdonald R. L., Ai J. & Yucel Y. H. *In vivo* imaging of lymphatic drainage of cerebrospinal fluid in mouse. Fluids Barriers CNS 10, 35, doi: 10.1186/2045-8118-10-35 (2013). 24360130 PMC3879644

[b49] Clarkson B. D. . CCR2-dependent dendritic cell accumulation in the central nervous system during early effector experimental autoimmune encephalomyelitis is essential for effector T cell restimulation *in situ* and disease progression. Journal of immunology 194, 531–541, doi: 10.4049/jimmunol.1401320 (2015). PMC4362728 25505278

[b50] Clarkson B. D. . Mapping the accumulation of co-infiltrating CNS dendritic cells and encephalitogenic T cells during EAE. Journal of neuroimmunology 277, 39–49, doi: 10.1016/j.jneuroim.2014.09.016 (2014). 25288303 PMC4250311

[b51] Serafini B., Rosicarelli B., Magliozzi R., Stigliano E. & Aloisi F. Detection of ectopic B-cell follicles with germinal centers in the meninges of patients with secondary progressive multiple sclerosis. Brain Pathol 14, 164–174 (2004). 15193029 10.1111/j.1750-3639.2004.tb00049.x PMC8095922

[b52] Magliozzi R., Columba-Cabezas S., Serafini B. & Aloisi F. Intracerebral expression of CXCL13 and BAFF is accompanied by formation of lymphoid follicle-like structures in the meninges of mice with relapsing experimental autoimmune encephalomyelitis. Journal of neuroimmunology 148, 11–23, doi: 10.1016/j.jneuroim.2003.10.056 (2004). 14975582

[b53] Magliozzi R. . Meningeal B-cell follicles in secondary progressive multiple sclerosis associate with early onset of disease and severe cortical pathology. Brain: a journal of neurology 130, 1089–1104, doi: 10.1093/brain/awm038 (2007). 17438020

[b54] Magliozzi R. . A Gradient of neuronal loss and meningeal inflammation in multiple sclerosis. Annals of neurology 68, 477–493, doi: 10.1002/ana.22230 (2010). 20976767

[b55] Howell O. W. . Meningeal inflammation is widespread and linked to cortical pathology in multiple sclerosis. Brain: a journal of neurology 134, 2755–2771, doi: 10.1093/brain/awr182 (2011). 21840891

[b56] Kooi E. J., Geurts J. J., van Horssen J., Bo L. & van der Valk P. Meningeal inflammation is not associated with cortical demyelination in chronic multiple sclerosis. Journal of neuropathology and experimental neurology 68, 1021–1028, doi: 10.1097/NEN.0b013e3181b4bf8f (2009). 19680141

[b57] Kuerten S. . Tertiary lymphoid organ development coincides with determinant spreading of the myelin-specific T cell response. Acta neuropathologica 124, 861–873, doi: 10.1007/s00401-012-1023-3 (2012). 22842876

[b58] Peters A. . Th17 cells induce ectopic lymphoid follicles in central nervous system tissue inflammation. Immunity 35, 986–996, doi: 10.1016/j.immuni.2011.10.015 (2011). 22177922 PMC3422678

[b59] Molnarfi N. . MHC class II-dependent B cell APC function is required for induction of CNS autoimmunity independent of myelin-specific antibodies. J Exp Med 210, 2921–2937, doi: 10.1084/jem.20130699 (2013). 24323356 PMC3865476

[b60] Hopken U. E. . CCR7 deficiency causes ectopic lymphoid neogenesis and disturbed mucosal tissue integrity. Blood 109, 886–895, doi: 10.1182/blood-2006-03-013532 (2007). 17018859

[b61] Halle S. . Induced bronchus-associated lymphoid tissue serves as a general priming site for T cells and is maintained by dendritic cells. J Exp Med 206, 2593–2601, doi: 10.1084/jem.20091472 (2009). 19917776 PMC2806625

[b62] Wendland M. . Lymph node T cell homeostasis relies on steady state homing of dendritic cells. Immunity 35, 945–957, doi: 10.1016/j.immuni.2011.10.017 (2011). 22195748

[b63] Chyou S. . Coordinated regulation of lymph node vascular-stromal growth first by CD11c+ cells and then by T and B cells. J Immunol 187, 5558–5567, doi: 10.4049/jimmunol.1101724 (2011). 22031764 PMC3221869

