# Supplementary material for: CCR7 deficient inflammatory Dendritic Cells are retained in the Central Nervous System
Source: Sci Rep. 2017 Feb 20;7:42856. doi: 10.1038/srep42856 (PMC5316931; doi:10.1038/srep42856)
Supplement: Supplementary Data File [file srep42856-s1.pdf]

Supplementary Data file:

CCR7 deficient inflammatory Dendritic Cells are retained in the Central Nervous System

Clarkson, Benjamin D, Alec Walker, Melissa G. Harris, Aditya Rayasam, Martin Hsu, Matyas Sandor, and Zsuzsanna Fabry

Figure S1. Comparison of costimulatory molecule expression and T cell activation capacity between CCR7 KO and WT BMDC.

A) Surface expression of costimulatory markers on WT and CCR7 KO BMDC upon stimulation with PBS (red) or LPS (blue) prior to intracerebral injection. **B)** Naïve MOG-specific CD4+ T cells were isolated and purified from 2D2 mice using MAGCellect naïve CD4+ T cell negative isolation kit (R&D systems). Purified 2D2 cells were briefly labeled with CFSE and then seeded at 20:1 with LPS-matured, MOG-pulsed WT or CCR7KO BMDC and incubated at 37C. After 5 days, cells were collected, stained with antibodies for CD4, Vβ11, and LFA-1, and acquired by flow cytometry. Mean percentage of 2D2 cells having undergone at least one round of proliferation (CFSE low) is plotted for each group. (n=3/group, in 3 experiments).

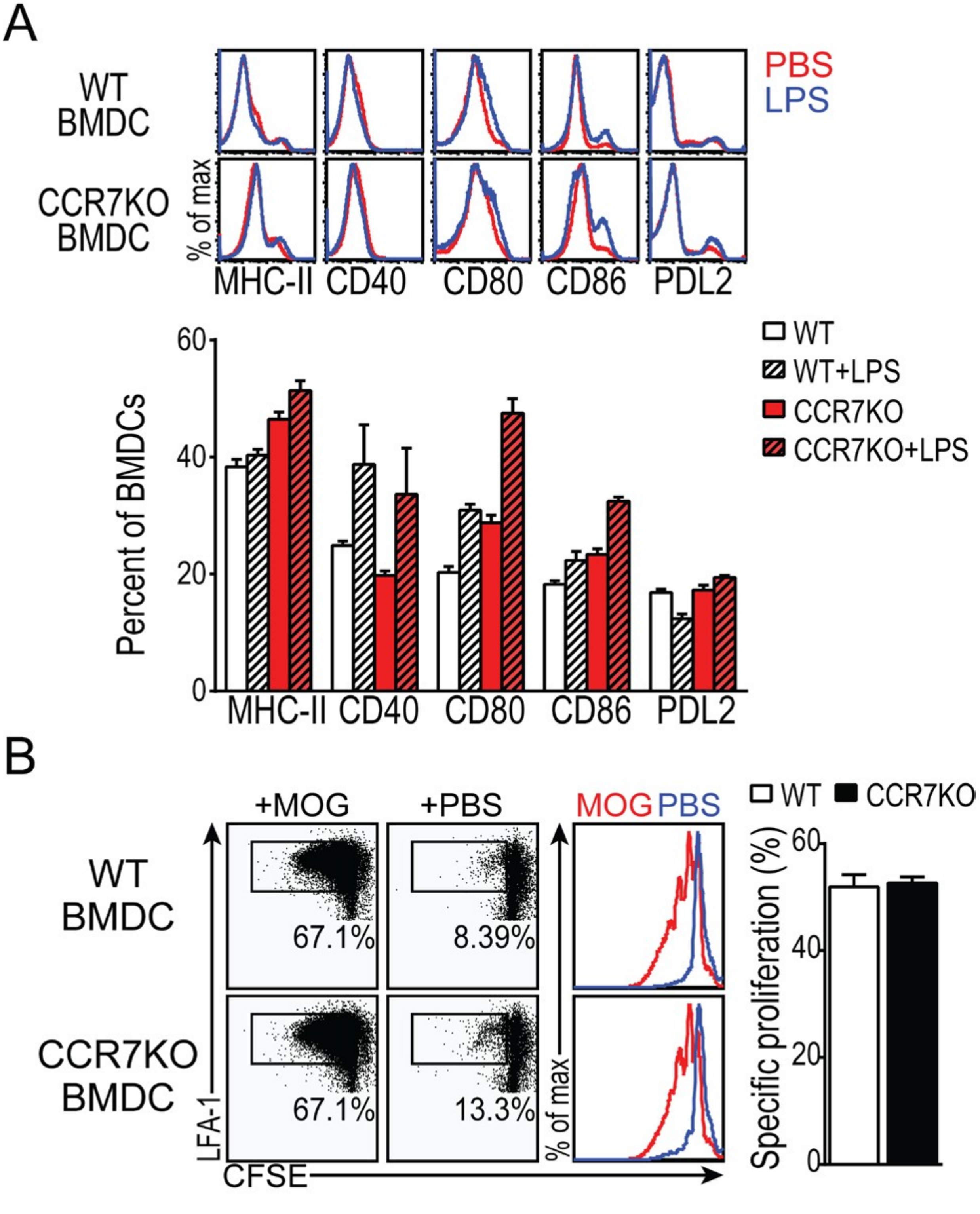
A
WT
BMDC
CCR7KO
BMDC
% of max
MHC-II CD40 CD80 CD86 PDL2
PBS
LPS
Percent of BMDCs
60
40
20
0
MHC-II CD40 CD80 CD86 PDL2
WT
WT+LPS
CCR7KO
CCR7KO+LPS
B
+MOG
+PBS
MOG PBS
WT
BMDC
CCR7KO
BMDC
67.1%
8.39%
67.1%
13.3%
LFA-1
CFSE
% of max
WT
CCR7KO
Specific proliferation (%)
60
40
20
0

Figure S2. I.C. injected fluorescent nanoparticles fail to accumulate in CLN DC in CCR7 KO mice compared to WT mice.

A) Column graphs show frequency of fluorescent red nanoparticle-containing CD11c-eYFP+ cells in deep CLN 1, 4, and 7 days following i.c. injection of nanoparticles with adjuvant (200 ng CCL2 and 150 ng LPS), **$p<0.01$, *$p<0.05$, student's t test. Gating of CD11c-eYFP+ cells is shown in **(B)**.

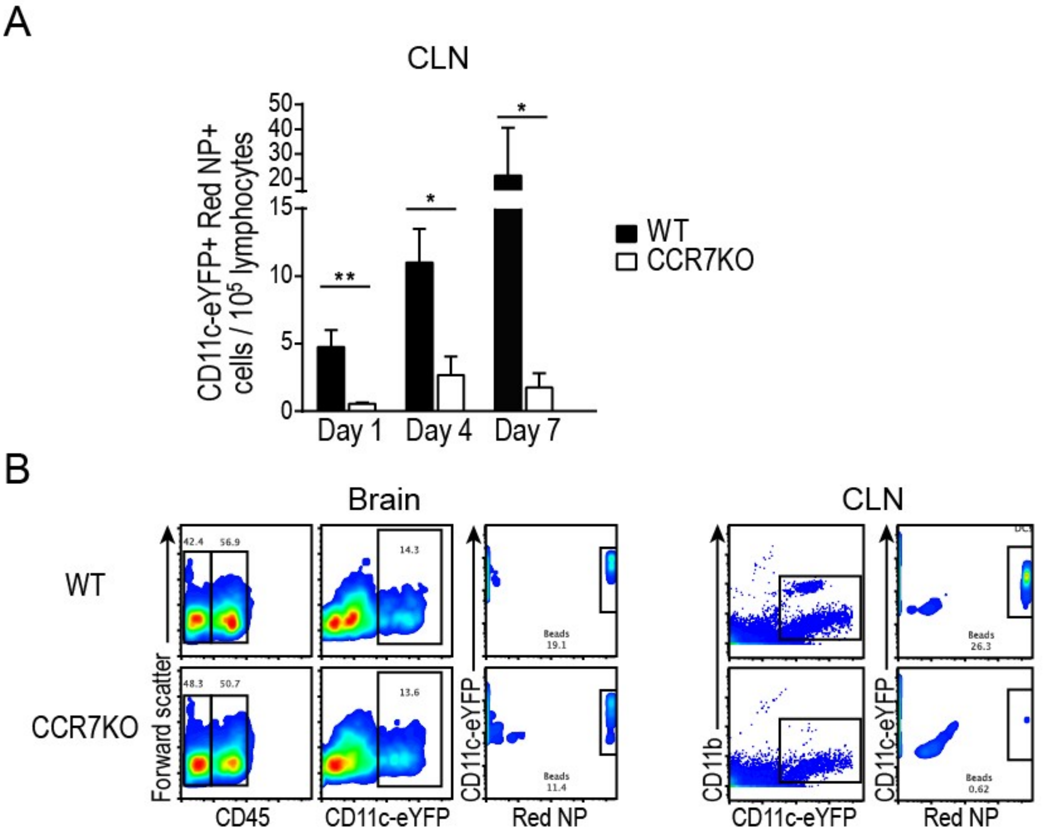
A
CLN
CD11c-eYFP+ Red NP+ cells / 10^5 lymphocytes
50
40
30
20
15
10
5
0
**
*
*
Day 1
Day 4
Day 7
WT
CCR7KO
B
Brain
CLN
WT
CCR7KO
42.4
56.9
14.3
Beads 19.1
Beads 26.3
48.3
50.7
13.6
Beads 11.4
Beads 0.62
Forward scatter
CD45
CD11c-eYFP
CD11c-eYFP
Red NP
CD11b
CD11c-eYFP
CD11c-eYFP
Red NP
